# Retraction Note: A synthetic dsRNA, as a TLR3 pathwaysynergist, combined with sorafenib suppresses HCC in vitro and in vivo

**DOI:** 10.1186/s12885-023-11135-3

**Published:** 2023-07-07

**Authors:** Yu-Yin Xu, Li Chen, Gui-Lan Wang, Jia-Ming Zhou, Yi-Xin Zhang, Yin-Ze Wei, Yuan-Yuan Zhu, Jing Qin

**Affiliations:** 1 Department of Pathological Anatomy, Nantong University, Nantong, China; 2 Department of Nephrology, Affiliated Hospital of Nantong University, Nantong, Jiangsu China; 3 Department of Pathology, Affiliated Tumor Hospital, Nantong University, Nantong, China; 4 Biomics (Nantong) Co., Ltd, Nantong, China

**Retraction Note: BMC Cancer 13, 527 (2013)**

10.1186/1471-2407-13-527

The Editor has retracted this article because it appears that some of the text and figures have been previously published by some of the same authors. Specifically:

- there is substantial text overlap with results section in [1]
- Figure 2B appears to present the same data as Fig. 1C in [1]
- It appears the same FACS plots have been presented in Fig. 3B (for srfn, poly (I:C) and control) as in Fig. 4A in [1]
- Figure 3C (sifn and Poly(I:C) ) and 3D appear to show the same images as those published previously in [1] in Fig. 3A

Author Yu-Yin Xu does not agree to this retraction. None of the other authors have responded to any correspondence from the editor or publisher about this retraction

## References

[1] Chen, L., Xu, Y., Zhou, J., Wu, Y., E, Q., Zhu, Y. “TLR3 dsRNA agonist inhibits growth and invasion of HepG2.2.15 HCC cells”. Oncology Reports 28.1 (2012): 200–206. 10.3892/or.2012.1791 22552584

